# Demographic histories of adaptively diverged riparian and non-riparian species of *Ainsliaea* (Asteraceae) inferred from coalescent analyses using multiple nuclear loci

**DOI:** 10.1186/1471-2148-12-254

**Published:** 2012-12-28

**Authors:** Yuki Mitsui, Hiroaki Setoguchi

**Affiliations:** 1 Faculty of Agriculture, Tokyo University of Agriculture, Funako 1737, Atsugi, Kanagawa, 243-0034, Japan; 2 Graduate School of Human and Environmental Studies, Kyoto University, Yoshida Nihonmatsu-cho, Sakyo-ku, Kyoto, 606-8501, Japan

**Keywords:** Adaptive divergence, Divergence time, Effective population size, Gene flow, Isolation-with-migration model, Riparian plants

## Abstract

**Background:**

Understanding demographic histories, such as divergence time, patterns of gene flow, and population size changes, in ecologically diverging lineages provide implications for the process and maintenance of population differentiation by ecological adaptation. This study addressed the demographic histories in two independently derived lineages of flood-resistant riparian plants and their non-riparian relatives [*Ainsliaea linearis* (riparian) and *A*. *apiculata* (non-riparian); *A*. *oblonga* (riparian) and *A*. *macroclinidioides* (non-riparian); Asteraceae] using an isolation-with-migration (IM) model based on variation at 10 nuclear DNA loci.

**Results:**

The highest posterior probabilities of the divergence time parameters were estimated to be *ca*. 25,000 years ago for *A*. *linearis* and *A*. *apiculata* and *ca*. 9000 years ago for *A*. *oblonga* and *A*. *macroclinidioides*, although the confidence intervals of the parameters had broad ranges. The likelihood ratio tests detected evidence of historical gene flow between both riparian/non-riparian species pairs. The riparian populations showed lower levels of genetic diversity and a significant reduction in effective population sizes compared to the non-riparian populations and their ancestral populations.

**Conclusions:**

This study showed the recent origins of flood-resistant riparian plants, which are remarkable examples of plant ecological adaptation. The recent divergence and genetic signatures of historical gene flow among riparian/non-riparian species implied that they underwent morphological and ecological differentiation within short evolutionary timescales and have maintained their species boundaries in the face of gene flow. Comparative analyses of adaptive divergence in two sets of riparian/non-riparian lineages suggested that strong natural selection by flooding had frequently reduced the genetic diversity and size of riparian populations through genetic drift, possibly leading to fixation of adaptive traits in riparian populations. The two sets of riparian/non-riparian lineages showed contrasting patterns of gene flow and genetic differentiation, implying that each lineage showed different degrees of reproductive isolation and that they had experienced unique evolutionary and demographic histories in the process of adaptive divergence.

## Background

Adaptation to diverse ecological habitats is a major force driving population differentiation, leading to the evolution of reproductive isolation, and ultimately speciation (Schluter, [1,2]; Coyne & Orr, [3]; Rundle & Nosil, [4]; Sobel *et al.*[5]). Theoretical studies suggest that populations in differential ecological habitats can develop reproductive isolation through divergent natural selection in a few thousand generations (Gavrilets & Vose, [6]) or even in dozens to hundreds of generations (Hendry *et al.*[7]). Recently, progress in molecular and population genetic techniques has allowed us to estimate the evolutionary and demographic histories of recently diverged populations or species reliably. For example, model-based simulation analysis, such as the isolation-with-migration (IM) model (Hey & Nielsen, [8]; Hey, [9]), can simultaneously estimate the divergence time, bidirectional gene flow since the initial split, and fluctuations in effective population sizes among recently diverged lineages based on the nucleotide variation at multiple loci. Estimates of these demographic parameters in ecologically divergent lineages provide important insights into evolutionary histories, population dynamics, and the maintenance of adaptive divergence.

The flood-resistant riparian plants referred to as rheophytes (van Steenis [10]) are remarkable examples of plant ecological adaptation. They are riparian specialists that inhabit riverbank habitats, where individuals are subjected to frequent submergence during intermittent floods after heavy rains. There are several advantages to using flood-resistant riparian plants to investigate the mechanisms of adaptive ecological divergence. First, the major selective force (flooding) and the resultant adaptive trait evolution (narrow, thick leaves) are explicit. To resist a strong flow of water, these plants typically evolve a narrow, thick leaf (Usukura *et al.*[11]; Imaichi & Kato, [12]; Kato, [13]; Tsukaya, [14]; Setoguchi & Kajimaru, [15]; Nomura *et al.*[16]), increasing mechanical toughness of the leaves (van Steenis, [10]). Second, flood-resistant riparian plants and their close non-riparian relatives with distinct phenotypes (round, broad leaves) are often found in parapatry across riverbank–forest floor transitions (van Steenis, [10]; Kato, [13]; Mitsui *et al.*[17]). The non-riparian relatives inhabit adjacent forest floor environments where flooding never reaches, providing opportunities to investigate divergence via divergent natural selection resulting from differential ecological habitats. Non-riparian relatives usually have much wider ranges than the riparian species, implying that most riparian lineages are derived from inland progenitors after pioneering in flood habitats in each locality (Kato, [13]). Third, most of these riparian taxa are considered to have evolved convergently in each region via local adaptation because they occupy separate taxonomic groups in angiosperms, as well as pteridophytes, which are found worldwide. Approximately 1,000 riparian vascular plant species have been reported (van Steenis [18]), and most (75%) are considered to be locally endemic with quite narrow ranges (Kato, [13]). Therefore, comparative analysis of adaptive divergence among independently evolved riparian lineages is possible.

**Figure 1:**
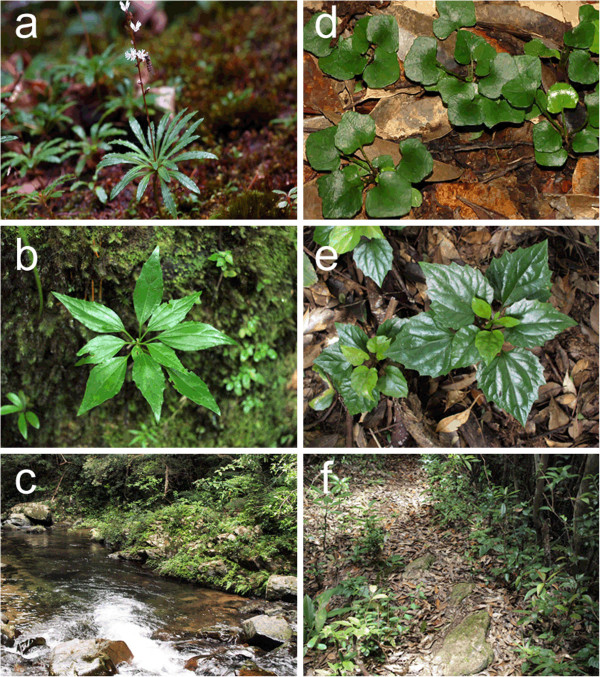
**Photos of the riparian species (a) *Ainsliaea linearis*, (b) *A. oblonga *and (c) their habitats, and photos of the inland species (d) *A. apiculata*, (e) *A. macroclinidioides *and (f) their habitats**.

**Figure 2:**
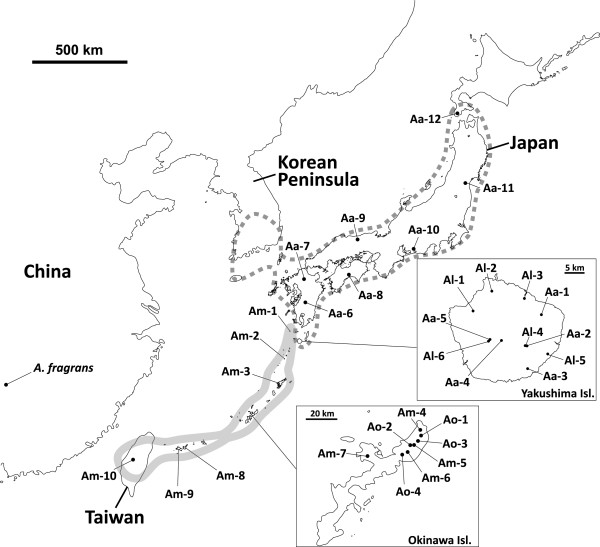
**The localities of the populations of *Ainsliaea linearis *(Al), *A. apiculata *(Aa), *A. oblonga *(Ao), and *A. macroclinidioides *(Am) sampled in this study.** Two riparian species, *A. linearis* and *A. oblonga*, are endemic to Yakushima Island and Okinawa Island, respectively. The distribution ranges of non-riparian species, *A. apiculata* and *A. macroclinidioides*, are represented by dotted and bold gray lines, respectively. Detailed information on these localities is provided in Additional file 3: Table S3.

This study investigated the evolutionary and demographic history of the adaptively diverged plants in *Ainsliaea* (Asteraceae); two independent lineages of flood-resistant perennial species, *Ainsliaea linearis* Makino and *A*. *oblonga* Koidz., and their closely related species distributed in the Japanese archipelago (Figure 1). *Ainsliaea linearis* is endemic to Yakushima Island, in the southern part of the Japanese archipelago (Figure 2). It is a riparian species that is strictly confined to rocky surfaces or crevices along riverbanks, where individuals are often subjected to flash floods after rains. *A*. *linearis* has developed narrow, streamlined leaves (Figure 1a). *Ainsliaea apiculata* Sch. Bip., the sister species of *A*. *linearis* (Mitsui & Setoguchi, [19]), is distributed across the Japanese archipelago, and Yakushima Island is the southernmost part of its range (Figure 2). *A*. *apiculata* has ovate or rounded leaves with shallow lobes and inhabits the floor or margins of deciduous or conifer forests (Figure 1d). Its range largely overlaps that of *A*. *linearis* on Yakushima Island, where the two species frequently grow in parapatry across riverbank–forestry transitions (Watanabe *et al.*[20]). A recent study using microsatellite DNA markers indicated that parapatric populations of *A*. *linearis* (synonym: *A*. *faurieana* Beauv.) and *A*. *apiculata* are isolated reproductively, mainly by divergent natural selection arising from their contrasting riparian and inland habitats, despite ongoing hybridization (Mitsui *et al.*[17]). Despite their distinct morphology and ecology, little genetic differentiation in some nuclear and chloroplast DNA regions has been detected within this monophyletic group (Mitsui *et al.*[21]). This implies that the adaptive divergence occurred over a relatively short evolutionary timescale.

Another riparian species, *Ainsliaea oblonga*, is endemic to Okinawa Island, part of the Ryukyu–Taiwan island chain, which is located between southeast China and mainland Japan (Figure 2). *A*. *oblonga*, like *A*. *linearis*, grows in flooded habitats. The leaves of *A*. *oblonga* are long and elliptical, and the base is cuneate, showing adaptive characteristics in flooded habitats (Koyama, [22]; Figure 1b). The closely related sister species *A*. *macroclinidioides* Hayata is widely distributed across the Ryukyu–Taiwan island chain (Mitsui & Setoguchi, [19]). It has ovate or rounded leaves with shallow lobes and inhabits the floor or margins of laurel forests (Figure 1e). The two species occur together in northern Okinawa Island. *A*. *oblonga* grows along riverbanks in gorges that are flooded regularly, while *A*. *macroclinidioides* grows on upper slopes and ridges. Although the two species are often distributed in contact (up to tens to hundreds of meters), they show clear segregation in flooded and forest floor habitats. In common garden experiments, the leaf shapes of the two species were stable, suggesting that the morphological characteristics are fixed genetically. In addition, viable, fertile hybrids with an intermediate leaf shape can be produced with artificial crossing (M. Mitsui, unpublished). In natural populations, however, intermediate phenotypes have not been seen, suggesting that the divergence of *A*. *oblonga* and *A*. *macroclinidioides* is likely maintained by ecological factors, such as habitat segregation that could lead to pre- or post-mating isolation.

We investigated the divergence time, significance and directions of gene flow, and effective population size differences among riparian (specialist, localized) and non-riparian (generalist, widespread) species in *Ainsliaea* (Asteraceae) using the IM model based on the multilocus datasets of nuclear gene sequences. The specific hypotheses addressed are the following. First, the initial events splitting the riparian and non-riparian species might be recent, despite their distinct morphological and ecological differences. Second, with adaptive divergence between the riparian and non-riparian populations, they could have exchanged genes since the initial split. The patterns of gene flow (bidirectional, unidirectional, or no gene flow) might have implications for population dynamics, the relative strength of reproductive isolation, and selective pressures between the ecologically divergent riparian/non-riparian lineages. Third, the riparian specialists could have less genetic variation and smaller effective population sizes than the non-riparian populations owing to genetic drift. Comparative analyses of the two riparian species with different origins might show common or different patterns of divergence, providing implications for the process and maintenance of adaptive divergence to highly disturbed flooded habitats in this group of taxa.

## Results

### Patterns of polymorphism and genetic divergence in riparian and non-riparian species

We sequenced 10 nuclear loci with a total length of 4502 bp from 70 individuals representing 6 populations of *A*. *linearis*, 12 of *A*. *apiculata*, 4 of *A*. *oblonga*, and 10 populations of *A*. *macroclinidioides*. The length of the aligned sequences for each locus ranged from 255 to 707 bp (Table 1). We found five insertion–deletion (indel) polymorphisms across all loci, but we did not include indels in the subsequent analyses. All sequences with different haplotypes (Additional file 1: Table S1) were deposited in the DNA Data Bank of Japan (DDBJ), with accession numbers AB601211–AB601418.

**Table 1:** Summary of nucleotide polymorphisms and genetic divergence of 10 loci for each monophyletic riparian and non-riparian species

			***A. linearis *(riparian)**					***A. apiculata *(non-riparian)**					**Divergence**	
	**Aligned size (bp)**	**Largest non-recombining block**	**No. of seqs.**					**No. of seqs.**						
**Locus**				***S***	***π***	***θ***	***Rm***		***S***	***π***	***θ***	***Rm***	***S***_**f**_	***D***_**XY**_
GA2ox1	707	642	24	3	0.0015	0.0011	0	48	4	0.0008	0.0011	0	0	0.0027
CHS	358	358	24	1	0.0011	0.0008	0	48	1	0.0011	0.0008	0	0	0.0011
GTF	382	240	24	0	0.0000	0.0000	0	48	5	0.0043	0.0000	1	1	0.0054
CPPS1	501	501	24	1	0.0006	0.0005	0	48	1	0.0000	0.0005	0	0	0.0016
A25	280	236	24	3	0.0033	0.0029	0	48	4	0.0030	0.0029	0	0	0.0038
A27	685	638	24	0	0.0000	0.0000	0	48	7	0.0022	0.0000	1	3	0.0082
B12	255	255	24	0	0.0000	0.0000	0	48	2	0.0003	0.0000	0	0	0.0005
D10	361	361	24	4	0.0013	0.0030	0	48	4	0.0041	0.0030	0	0	0.0030
D13	504	504	24	3	0.0017	0.0016	0	48	1	0.0011	0.0016	0	4	0.0105
D22	469	235	24	0	0.0000	0.0000	0	48	3	0.0022	0.0000	2	0	0.0033
Mean	450	397	24	1.5	0.0009	0.0010	0	48	3.2	0.0019	0.0010	0.4	0.8	0.0040
			***A. oblonga *(riparian)**					***A. macroclinidioides *(non-riparian)**					**Divergence**	
**Locus**	**Aligned size (bp)**	**Largest non-recombining block**	**No. of seqs.**	***S***	***π***	***θ***	***Rm***	**No. of seqs.**	***S***	***π***	***θ***	***Rm***	***S***_**f**_	***D***_**XY**_
GA2ox1	707	505	24	4	0.0007	0.0015	0	28	8	0.0033	0.0029	1	0	0.0022
CHS	358	358	24	1	0.0015	0.0008	0	28	3	0.0017	0.0022	0	0	0.0018
GTF	382	132	24	1	0.0009	0.0007	0	28	4	0.0043	0.0027	1	0	0.0038
CPPS1	501	501	24	1	0.0006	0.0005	0	28	1	0.0001	0.0005	0	0	0.0004
A25	280	222	24	2	0.0015	0.0018	0	28	4	0.0050	0.0035	1	0	0.0040
A27	685	434	24	10	0.0068	0.0039	3	28	11	0.0076	0.0041	3	0	0.0073
B12	255	255	24	1	0.0017	0.0011	0	28	1	0.0017	0.0010	0	0	0.0016
D10	361	195	24	5	0.0042	0.0037	1	28	4	0.0032	0.0029	2	0	0.0043
D13	504	421	24	5	0.0030	0.0026	1	28	6	0.0048	0.0030	0	0	0.0047
D22	469	302	24	6	0.0031	0.0034	0	28	6	0.0042	0.0033	2	0	0.0053
Mean	450	333	24	3.6	0.0024	0.0020	0.5	28	4.8	0.0036	0.0026	1.0	0	0.0035

*S*, number of segregating (polymorphic) sites.

π, average number of pairwise nucleotide differences per site.

*θ*, Watterson's estimator of *θ* per base pair.

*Rm*, estimate of the minimum number of recombination events.

*S*_f_, number of segregating sites that are fixed between the two speices.

*D*_XY_, average proportion of nucleotide differences between species.

The sequence polymorphisms and divergence between each monophyletic pair of riparian and non-riparian species (*i.e.*, between *A*. *linearis* and *A*. *apiculata* and between *A*. *oblonga* and *A*. *macroclinidioides*) are compared in Table 1. For *A*. *macroclinidioides*, the southern populations were excluded from the analyses because they clustered as distinct lineages in the phylogenetic analyses (see the results below). The genetic divergence between riparian and inland species was fairly small, and most of the polymorphisms were shared between them. The fixed nucleotide differences between *A*. *linearis* and *A*. *apiculata* averaged 0.8 per locus, while no fixed difference was observed between *A*. *oblonga* and *A*. *macroclinidioides*. We found that the riparian species tended to have fewer polymorphic sites and recombination events, and lower nucleotide diversities than the inland sister species.

None of the loci showed a consistently significant deviation from neutral expectations for Tajima’s *D* or Fu and Li’s D* and F* statistics, except the A27 locus for *A*. *oblonga* and *A*. *macroclinidioides* (Additional file 2: Table S2). For this locus, *A*. *oblonga* and *A*. *macroclinidioides* showed significant deviation from neutral expectations in all three tests. Since Tajima’s *D*-statistic is sensitive to population structure and demography (Tajima, [23]), we performed a multi-locus Hudson, Kreitman & Aguad (HKA) test, which is robust to population structure and demography. When using one sequence of *A*. *fragrans*, which occurs in southeast China, as an outgroup, we did not detect a significant departure from the equilibrium model for any of the comparisons between each species and the outgroup (*χ*^2^ = 3.09, *P* > 0.66 for *A*. *linearis*; *χ*^2^ = 2.04, *P* > 0.63 for *A*. *apiculata*; *χ*^2^ = 2.22, *P* > 0.64 for *A*. *oblonga*; and *χ*^2^ = 1.53, *P* > 0.53 for *A*. *macroclinidioides*). None of the 10 loci showed evidence of being under selection using all of the statistics for the species examined, and we used all loci for the subsequent analyses. Note that inclusion or exclusion of the A27 locus in the analyses did not significantly change the results.

### Phylogenetic relationships and population structure

**Figure 3:**
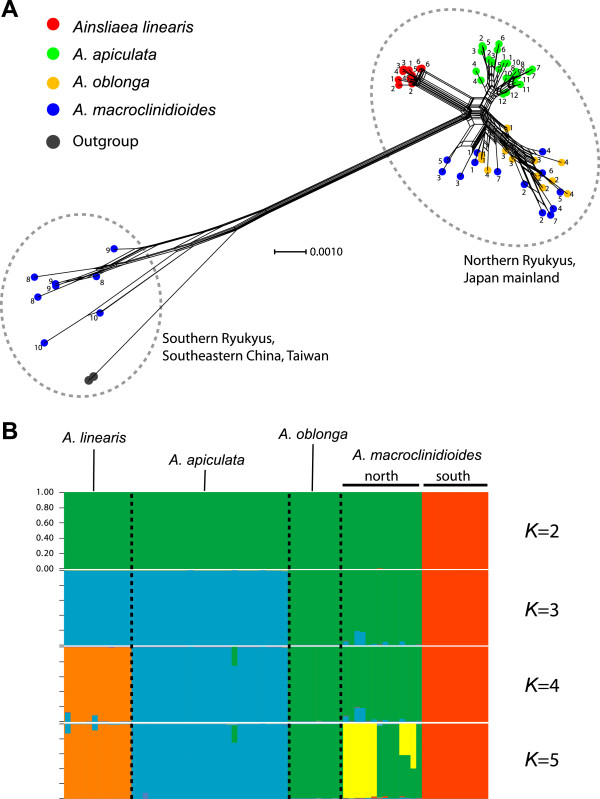
**(A) Phylogenetic network of the four species and outgroup of *Ainsliaea *reconstructed with the NeighborNet method.** The network is based on the combined sequences of 10 nuclear gene loci (total length 4502 bp) from 72 individuals, including two outgroup individuals. Each individual is numbered according to its locality. (**B**) Assignments of individuals to clusters (*K* = 2–5) for the four species inferred by STRUCTURE. The clustering pattern was highly consistent across 20 independent runs for each cluster, and one of the results is shown.

The phylogenetic network reconstructed using NeighborNet analysis showed that the populations of the riparian species *A*. *linearis* formed a cluster (Figure 3A). Conversely, the populations of the other riparian species, *A*. *oblonga*, were not separated genetically from the non-riparian relative, *A*. *macroclinidioides*. The genetic admixture in *A*. *oblonga* and *A*. *macroclinidioides* was also indicated in the STRUCTURE analysis (Figure 3B). The most likely number of clusters was four when the Δ*K* statistic of Evanno *et al.*[24] was applied (Δ*K* = 239.8). When *K* = 4, *A*. *linearis* and *A*. *apiculata* comprised different clusters, while *A*. *oblonga* and *A*. *macroclinidioides* were not separated from each other. The genetic admixture in the STRUCTURE analysis could reflect recent introgression between the two species (Duchesne & Turgeon, [25]).

By contrast, the phylogenetic and STRUCTURE analyses showed that the *A*. *macroclinidioides* populations exhibited distinct genetic structuring with regard to the northern (populations Am1–7) and southern (populations Am8–10) regions of the Ryukyu–Taiwan island chain, suggesting that *A*. *macroclinidioides* is a non-monophyletic group. The southern populations showed close relationships with Chinese outgroup species. There was no evidence of within-species geographical structuring for the four species, with the exception of *A*. *macroclinidioides*. Therefore, the southern populations of *A*. *macroclinidioides* were excluded from the riparian/non-riparian comparisons.

### Estimating demographic parameters

The maximum-likelihood estimates (MLEs) and 90% highest posterior density (HPD) of the population genetic parameters estimated by IM are shown in Table 2. The parameter estimates were scaled using the geometric mean of the per locus mutation rate to make the parameters more easily interpretable. The analyses were conducted separately for each monophyletic pair of riparian and non-riparian species.

**Table 2:** Maximum-likelihood estimates (MLEs) and the 90% highest posterior density (HPD) intervals of model parameters from IMa analyses

	***θ***_**1**_	***θ***_**2**_	***θ***_**A**_	***m***_**1**_	***m***_**2**_	***t***	**N**_**1**_	**N**_**2**_	**N**_**A**_	**m**_**1**_	**m**_**2**_	**2N**_**1**_**m**_**1**_	**2N**_**2**_**m**_**2**_	**T (year)**
**(1) *A. linearis vs. *(2) *A. apiculata***														
MLE	0.095	0.263	0.718	2.665	0.388	0.065	8643	23831	65189	0.007	0.001	0.127	0.051	24782
HPD90Lo	0.040	0.132	0.004	0.655	0.005	0.018	4010	12483	9709	0.000	0.000	0.013	0.000	9074
HPD90Hi	0.207	0.454	7.171	6.538	1.802	8.628	21473	45014	704721	0.023	0.008	0.677	0.409	3131867
**(1) *A. oblonga vs. *(2) *A. macroclinidioides *(north)**														
MLE	0.053	0.492	0.772	19.827	10.165	0.025	4579	44819	70084	0.057	0.023	0.526	2.499	9074
HPD90Lo	0.022	0.223	0.006	4.777	2.925	0.005	1902	20152	501	0.009	0.005	0.053	0.326	1815
HPD90Hi	0.170	0.912	9.641	48.573	23.715	9.985	15007	82918	873072	0.168	0.063	4.118	10.810	3624008
**(1) North *vs. *(2) South**														
MLE	0.436	0.585	0.158	0.005	0.208	1.605	60536	81181	21950	0.000	0.000	0.001	0.061	890679
HPD90Lo	0.264	0.354	0.013	0.005	0.035	0.545	36578	49127	1742	0.000	0.000	0.001	0.006	302442
HPD90Hi	0.668	0.927	12.359	0.215	0.488	26.835	92622	128566	1714557	0.001	0.001	0.072	0.226	14891815

N_1_, N_2_, N_A_: effective population sizes of population 1, 2 and ancestral. *θ*=4Nu.

m_1_: the rate of gene flow from population 2 to population 1 per gene per generation. *m*_1_=m_1_/u.

m_2_: the rate of gene flow from population 1 to population 2 per gene per generation. *m*_2_=m_2_/u.

2N_1_m_1_: the effective rate of gene flow from population 2 to population 1 per generation.

2N_2_m_2_: the effective rate of gene flow from population 1 to population 2 per generation.

Each value is the average of three independent runs of the simulations. Demographic quantities, N_1_, N_2_, N_A_, m_1_, m_2_, and T, were converted based on a mutation rate (u) of 7.49 × 10^–9^ substitutions/site/year.

**Figure 4:**
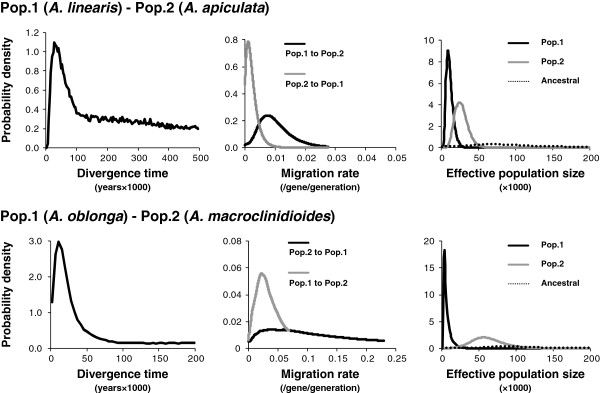
**Marginal distribution of the posterior probability of divergence time (years × 1000), migration rates per gene per generation, and effective population sizes (× 1000) estimated by IMa analyses conducted for each pair of riparian and non-riparian species**.

In the divergence parameter estimation the three independent runs consistently showed a sharp peak in each dataset, however, the marginal posterior probability distribution of the parameter did not drop to zero when sufficiently high values were reached. The MLEs of the divergence parameters were *ca*. 25,000 years ago (90% HPD 9074–3,131,867 years ago) for *A*. *linearis* and *A*. *apiculata* and *ca*. 9000 years ago (90% HPD 1815–3,624,008 years ago) for *A. oblonga* and *A*. *macroclinidioides* (Figure 4). The estimated wide confidence intervals were likely due to small data set that may not be enough to achieve convergence.

The strict isolation model in which no gene flow occurred during the divergence of the two sets of riparian and inland species was rejected by likelihood ratio tests for both pairs (Table 3; the likelihoods of the models with *m*_1_ = 0 *m*_2_ = 0 were significantly low than that of the full model). The gene flow between riparian *A*. *linearis* and non-riparian *A*. *apiculata* was estimated to be unidirectional; the gene flow from *A*. *apiculata* to *A*. *linearis* was significantly greater than zero (m_1_ = 0.007, 90% HPD 0.000–0.023), while there was no evidence of gene flow in the opposite direction (m_2_ = 0.001, 90% HPD 0.000–0.008; Table 2). The population migration rates was 0.127 (2N_1_m_1_) from *A*. *apiculata* to *A*. *linearis* and 0.051 (2N_2_m_2_) in the opposite direction.

**Table 3:** Tests of significance of migration and population size differences

	***A. linearis *(1) -**				***A. oblonga *(1) -**				**North (1) - South (2)**			
	***A. apiculata *(2)**				***A. macroclinidioides *(north) (2)**							
**Model**	**log(P)**	**df**	**2LLR**	***P***	**log(P)**	**df**	**2LLR**	***P***	**log(P)**	**df**	**2LLR**	***P***
*θ*_A_*θ*_1_*θ*_2_*m*_1_ = *m*_2_	2.4387	1	6.4799	**0.011**	−3.7251	1	8.0867	**0.004**	4.9933	1	2.6006	0.107
*θ*_A_*θ*_1_*θ*_2_*m*_1_ *m*_2_=0	5.6746	1	0.008	0.929	−2.4051	1	5.4467	**0.020**	2.1984	1	7.7043	**0.006**
*θ*_A_*θ*_1_*θ*_2_*m*_1_=0 *m*_2_	2.4387	1	6.4799	**0.011**	−1.9985	1	4.6336	**0.031**	5.7494	1	0.0043	0.948
*θ*_A_*θ*_1_*θ*_2_*m*_1_=0 *m*_2_=0	2.4394	2	6.4784	**0.039**	−3.3128	2	7.2621	**0.026**	2.1981	2	7.7057	**0.021**
*θ*_A_*θ*_1_=*θ*_2_*m*_1_ *m*_2_	1.4532	1	8.4509	**0.004**	−2.6265	1	5.8896	**0.015**	5.5204	1	1.3799	0.240
*θ*_A_=*θ*_1_=*θ*_2_*m*_1_ *m*_2_	0.2299	2	10.8975	**0.004**	−6.3303	2	13.2971	**0.001**	4.156	2	1.4858	0.476
*θ*_A_*θ*_1_=*θ*_2_*m*_1_=*m*_2_	1.4449	2	8.4674	**0.014**	−10.614	2	21.8637	**0.000**	4.7742	2	4.5953	0.100
*θ*_A_*θ*_1_=*θ*_2_*m*_1_=0 *m*_2_=0	−5.1614	3	21.68	**0.000**	−145.77	3	292.18	**0.000**	0.9275	3	10.8436	**0.013**
*θ*_A_=*θ*_1_=*θ*_2_*m*_1_=*m*_2_	0.178	3	11.0012	**0.012**	−10.805	3	22.2464	**0.000**	3.3505	3	4.8739	0.181
*θ*_A_=*θ*_1_=*θ*_2_*m*_1_=0 *m*_2_=0	−34.912	4	81.1808	**0.000**	−279.85	4	560.328	**0.000**	−1.3107	4	18.0984	**0.001**
*θ*_A_=*θ*_1_*θ*_2_*m*_1_ *m*_2_	1.6998	1	7.9577	**0.005**	−3.4594	1	7.5554	**0.006**	4.8486	1	0.3983	0.528
*θ*_A_=*θ*_1_*θ*_2_*m*_1_=*m*_2_	0.7965	2	9.7642	**0.008**	−4.5652	2	9.7669	**0.008**	4.0603	2	2.9642	0.227
*θ*_A_=*θ*_1_*θ*_2_*m*_1_=0 *m*_2_=0	−34.889	3	81.1346	**0.000**	−270.87	3	542.386	**0.000**	−1.0241	3	12.4439	**0.006**
*θ*_1_*θ*_A_=*θ*_2_*m*_1_ *m*_2_	1.5984	1	8.1604	**0.004**	−3.9078	1	8.4522	**0.004**	4.6988	1	0.536	0.464
*θ*_1_*θ*_A_=*θ*_2_*m*_1_=*m*_2_	0.4587	2	10.4398	**0.005**	−4.8235	2	10.2835	**0.006**	4.0149	2	3.1385	0.208
*θ*_1_*θ*_A_=*θ*_2_*m*_1_=0 *m*_2_=0	−25.328	3	62.0126	**0.000**	−11.575	3	23.7873	**0.000**	1.1371	3	8.6762	**0.034**

16 nested models (no migration and equal population size) were compared to the full model (significant migration and different population sizes; *θ*_A_*θ*_1_*θ*_2_*m*_1_ *m*_2_).

“log(P)” is the posterior probability of the model given data, “2LLR” = 2 × (Log(P)nested model-Log(P)full model), “df” is the difference in number of parameters between nested and full model, and the *P*-value is the probability of achieving the test statistic (2LLR) by chance under the null model. The models with *P* < 0.05 represent rejection of the models and they are indicated in boldface. *θ*_1_, *θ*_2_, *θ*_A_ are effective population sizes of population (1), population (2), and ancestral population, respectively. *m*_1_ and *m*_2_ are the gene flow from (2) to (1) and (1) to (2), respectively.

More gene flow was detected between riparian *A*. *oblonga* and non-riparian *A*. *macroclinidioides*, although the probability distributions did not show sharp peaks (Figure 4). Both directions of gene flow were significantly greater than zero (Table 3). The gene flow from non-riparian *A*. *macroclinidioides* to riparian *A*. *oblonga* was also higher (m_1_ = 0.057, 90% HPD 0.009–0.168) than in the opposite direction (m_2_ = 0.023, 90% HPD 0.005–0.063; Table 2). However, the population migration rates showed the opposite results; from *A*. *macroclinidioides* to *A*. *oblonga* (2N_1_m_1_) = 0.526 and that in the opposite direction (2N_2_m_2_) was 2.499, suggesting that the amount and direction of gene flow between the two sets of riparian/non-riparian lineages differed in each group.

We found that the effective populations of riparian species were significantly smaller than those of non-riparian species (Tables 2 and 3, Figure 4). The estimated effective population size of *A*. *linearis* was 8,643 (90% HPD 4010–21,473), nearly one-third that of *A*. *apiculata* (*ca*. 23,800; 90% HPD 12,483–45,014). The population size difference between *A*. *oblonga* and *A*. *macroclinidioides* was more remarkable. The riparian *A*. *oblonga* had the smallest effective population (4,579; 90% HPD 1902–15,007), nearly one-tenth that of the northern lineage of *A*. *macroclinidioides* (44,819; 90% HPD 20,152–82,918). The effective sizes of the ancestral populations tended to be larger than the descendent populations, although the posterior probabilities had wide, flat distributions. This significant reduction in the population size of riparian species is consistent with the relatively low genetic diversity compared with the non-riparian species.

## Discussion

### Origins of the two flood-resistant species of *Ainsliaea*

According to our estimation of demographic parameters using the IM model, the initial splits of the flood-resistant riparian plants in *Ainsliaea* might have occurred over short evolutionary timescales. Given the wide confidence intervals, however, there is uncertainty in the divergence time estimates. Such wide intervals were likely due to relatively small data set. The previous studies showed that the divergence time parameter tended not to reach to convergence for the recently diverged populations or taxa (Hey *et al.*[26]; Ikeda *et al.*[27]). The riparian and non-riparian relatives showed fairly low genetic differentiation that is consistent with the previous study using some nuclear and chloroplast DNA sequences (Mitsui *et al.*[21]). Based on the fact that the riparian/non-riparian species can hybridize and produce fertile progenies, our results support recent origins and rapid phenotypic evolution via adaptation to flooded habitats in this group of taxa. These findings are consistent with postulates that ecological differences can drive the evolution of phenotypes and partial reproductive barriers within short evolutionary timescales (Gavrilets & Vose, [6]). In animals, there is increasing evidence of recent species diversification via ecological adaptation (*e.g.*, Verheyen *et al.*[28]; Aguirre *et al.*[29]; Peccoud *et al.*[30]), but there is little evidence of this in plant species. Our study presents a potential case of rapid speciation in plants arising through adaptation to different abiotic environments.

The two riparian species showed different levels of genetic divergence from their non-riparian sister species. *A*. *linearis* and *A*. *apiculata*, which coexist on Yakushima Island, comprised distinct genetic clusters in the phylogenetic analysis, suggesting that they have accumulated genetic differentiation since their split. By contrast, the riparian species *A*. *oblonga* and its sister species *A*. *macroclinidioides*, which coexist on Okinawa, had no fixed nucleotide differences, and they were not clustered separately. A previous study demonstrated the origin of the flood-resistant riparian species *Solenogyne mikadoi*, an endemic species in the Ryukyu Islands (Nakamura *et al.*[31]). It showed that the divergence between *Solenogyne mikadoi* and the other *Solenogyne* species with disjunct distributions in southeastern Australia dated back several million years. This indicates that the riparian *Solenogyne mikadoi* is a relict species that had diverged from its non-riparian relatives in ancient times, following geographical isolation through range shifts and local extinction. On Okinawa Island the two riparian species, *A. oblonga* and *S. mikadoi*, occur in flooded riversides. Our results indicate that the existing taxa of flood-resistant plants have various evolutionary histories.

### Evidence of historical gene flow between riparian/non-riparian species

Our IM simulations rejected the strict isolation model of population divergence and provided evidence of historical gene flow between riparian and non-riparian species. Recent coalescent-based analyses—such as the IM model—have been used to distinguish between the effects of shared ancestral polymorphism and gene flow on the level of genetic differentiation (Hey, [32]). The likelihood ratio test indicated unidirectional gene flow from non-riparian *A*. *apiculata* to riparian *A*. *linearis*. One possible explanation of the unidirectional gene flow from riparian to non-riparian species is that the genes of riparian species are more difficult to move to non-riparian species inhabiting forest floor habitats. The nucleotide diversity of the riparian species was low, suggesting that their genomic components have undergone selection for specific types that have greater fitness in riparian habitats, but lower fitness in forest floor habitats. Although the statistical test suggested the existence of gene flow, the population migration rates (2Nm) between *A*. *linearis* and *A*. *apiculata* were below 1.0 for both directions, suggesting that the riparian/non-riparian species have been isolated genetically and are well-differentiated (Wright [33]).

By contrast, another set of riparian/non-riparian species, *A*. *oblonga* and *A*. *macroclinidioides*, was estimated to have exchanged genes at a relatively high rate since their divergence. The population migration rate from riparian *A*. *oblonga* to non-riparian *A*. *macroclinidioides* was 2.499 and that in the opposite direction was 0.526, suggesting that there was sufficient gene flow to prevent genetic differentiation between the two species. In the STRUCTURE analysis, the genetic admixture and absence of population clustering in the two species also indicate relatively recent gene flow (Duchesne & Turgeon, [25]). The greater amount of unidirectional gene flow from riparian *A*. *oblonga* to non-riparian *A*. *macroclinidioides* might be due to the difference in the strength of selective pressures compared to the other riparian/non-riparian pair: *A*. *linearis* and *A*. *apiculata*. Yakushima Island, where *A*. *linearis* and *A*. *apiculata* occur, is characterized by very high rainfall, up to 10,000 mm/year (Takahara & Matsumoto, [34]), leading to the development of fast-flowing rivers that often cause flooding after rains. It thus seems likely that divergent selection arising from riparian/non-riparian habitats is relatively stronger on Yakushima Island than Okinawa Island. The riparian/non-riparian species in each system can hybridize each other and they are reproductively isolated mainly by ecological divergence (Mitsui *et al.*[17]). The contrasting patterns of gene flow between the two riparian/non-riparian species pairs may be attributed to the different evolutionary history of divergence, reflecting different level of adaptive divergence in each system.

The gene flow between the northern and southern lineages of *Ainsliaea* was estimated to be nearly zero (m_1_ and m_2_ = 0.000), indicating that these regionally subdivided populations have long been strictly isolated. The estimated date of their divergence, *ca*. 0.8 mya, is congruent with the geological history that the Ryukyu–Taiwan island arc had separated from the nearby continent (Kimura, [35]). Notably, morphological differentiation cannot be recognized between these allopatric populations of *A*. *macroclinidioides* (Y. Mitsui, unpublished). Overall, the contrasting patterns of divergence (*i.e.*, recent divergence among morphologically and ecologically distinct species *vs.* ancient population divergence within a single species) highlight the relative importance of adaptation to new ecological habitats for phenotypic evolution and speciation in this group of taxa.

### Levels of genetic diversity and population sizes

We found lower genetic diversity and significantly smaller effective population sizes in riparian populations compared to the non-riparian populations. Flood-resistant riparian plants of various angiosperm and pteridophyte taxa have diversified globally (van Steenis [10,18]). The ranges of most riparian species are confined to fairly small areas, while the closely related non-riparian species have broad ranges. Therefore, in many cases, locally endemic riparian species are expected to have arisen from inland ancestral populations by adapting to flooding habitats. According to this assumption, our finding of lower genetic diversity and significantly smaller populations in riparian species might be a consequence of genetic drift during the process of adaptation to flooded habitats. During the process of local adaptation, natural selection might cause population declines and increase the potential for local extinction (Reznick and Ghalambor [36]). Consequently, founder populations are susceptible to genetic drift at the time of founding. Indeed, the effective population size of *A*. *oblonga*, which is thought to have speciated quite recently, was the lowest of the four species studied. Presently, the riparian species *A*. *linearis* occurs only on Yakushima Island and forms abundant metapopulations in the river systems (Mitsui *et al.*[37]). *A*. *oblonga* is also thought to maintain sufficiently large populations to prevent bottleneck effects that reduce genetic diversity (Y. Mitsui, personal observation). Given that the plants in riparian flooded habitats suffer severe selective pressures, the invasion of flooded habitats is likely achieved by small numbers of founders, leading to reduced genetic diversity and effective population sizes through genetic drift. The IM model assumes the absence of a population structure in the ancestral population and the sudden split, thus the model can be viewed as a simple case of divergence. In general, the effective population sizes of the descendant populations are well estimated, but the estimates of N_A_ are reported to be sensitive to model misspecification (Becquet & Przeworski, [38]). Thus, further investigations using other models of divergence with larger DNA data sets are needed to address the detailed demography.

## Conclusions

This study demonstrated the recent origin of flood-resistant riparian plants, which are remarkable examples of plant ecological adaptation to abiotic environments. The population demographic analyses showed evidence of historical gene exchange between riparian/non-riparian species with parapatric distribution. The two independently evolved riparian species showed reduced genetic diversity and effective population sizes, implying that strong natural selection by flooding frequently reduced diversity of riparian populations. The small sizes of effective populations of riparian species could have promoted the fixation of adaptive trait divergence in highly disturbed flooded habitats. Our results presented a potential case of rapid population differentiation and speciation by ecological adaptation in plants, and adaptive divergence between riparian and non-riparian species has been achieved in the face of gene flow.

## Methods

### Sample collections

For the riparian species, six populations of *A. linearis* on Yakushima Island and four populations of *A. oblonga* on Okinawa Island were collected (Figure 2). Each population was located in a different river system on each island. Non-riparian species comprised 12 populations of *A. apiculata* from the Japanese archipelago, which included five populations from Yakushima Island and 10 populations of *A. macroclinidioides* from the Ryukyu Islands and Taiwan, which included four populations from Okinawa Island. In total, 70 individuals of the four species were sampled across the distribution range. The localities and geographical ranges of the samples are shown in Additional file 3: Table S3. Total DNA was extracted from fresh or silica gel-dried leaves using the CTAB method based on Doyle & Doyle [39].

### PCR amplification and sequencing of nuclear loci

We determined the sequences of 10 nuclear loci. Three regions, GA2ox1, GTF, CPPS1, were amplified using primers originally developed based on the sequences of Asteraceae and a related family, which were obtained from the Compositae Genome Project Database (CGPDB: http://www.cgpdb.ucdavis.edu/) and GenBank database. CHS region was amplified using primers developed by Alvarez *et al.*[40], previously reported as single-copy genes. Six regions, A25, A27, B12, D10, D13, D22, were amplified using primers developed by Chapman *et al.*[41], which were universally applicable to the Asteraceae family. Locus information and a list of the primers used in the subsequent analyses are shown in Table 4.

**Table 4:** Locus information and list of the primers used in this study

**Locus**	**Best BLAST hit**	**E-value**	**Species**	**Gene**	**Primer name**	**Sequences 5'-3'**	**Primer source**
GA2ox1	AB031206	1.00E-104	*Lactuca sativa*	gibberellin 2-oxidase 1	GA2ox1 Ai2 F	GACCAAGCGTGATTTACTCTG	Developed for this study
					GA2ox1 R	TTCTCGCTCAATGGTGGTCCT	Developed for this study
CHS	X91343	8.00E-11	*Leibnitzia anandria*	chalcone synthase	1266 F	ATCACCCACCTCATCTTCTGCAC	Álvarez *et al.*[40]
					1990 R	TCCAAAAGATCGAGTTCCAGTC	Álvarez *et al.*[40]
GTF	AB070746	5.00E-06	*Vigna angularis*	glucosyltransferase-3	GTF F	ACCAGATGCACCCTATTCATCT	Developed for this study
					GTF R	AAAGCGTGGTGGTGCTGATT	Developed for this study
CPPS1	AF034545	1.00E-32	*Stevia rebaudiana*	copalyl pyrophosphate synthase (Cpps1)	CPPS1 F	GAKGGAGAGATAACTGTATC	Developed for this study
					CPPS1 R	GGKTYCGTCTTGCATAGATT	Developed for this study
A25	EF519751	1.00E-18	*Carthamus oxyacanthus*	Unknown	A25 F	TTGCATGSTCTTATCAGTCC	Chapman *et al.*[41]
					A25 R	GAAGABCCCATCCARCAGAAGAG	Chapman *et al.*[41]
A27	EF519770	4.00E-11	*Carthamus oxyacanthus*	Unknown	A27 F	CTTGCAWTGAATGTCATGTGGAAG	Chapman *et al.*[41]
					A27 R	GCTCCCCARCATTTCA	Chapman *et al.*[41]
B12	EF519836	3.00E-68	*Carthamus lanatus*	Unknown	B12 F	CAAGTGGCTGCAGCCATGGG	Chapman *et al.*[41]
					B12 R	ACATCRGGMACCATTCCWCCGGTGT	Chapman *et al.*[41]
D10	HM640003	5.00E-15	*Medicago truncatula*	somatic embryogenesis receptor kinase 5 (SERK5)	D10 F	GATTGCTYGTTTATCCCTACATGG	Chapman *et al.*[41]
					D10 R	ATATTTGCAGCTTTCACATC	Chapman *et al.*[41]
D13	No hit	-	-	Unknown	D13 F	ATGTCAGGTTTTGGRCAYCGTGT	Chapman *et al.*[41]
					D13 R	CCAGARTAGAAATCAACATTYGGGTAC	Chapman *et al.*[41]
D22	EF484030	5.00E-19	*Carthamus tinctorius*	Unknown	D22 F	CGHAGAACTCCAGCTGAA	Chapman *et al.*[41]
					D22 R	GCTTCTTCTTGCCTGATGCT	Chapman *et al.*[41]

### Haplotype determination

For those sequences that contained more than one heterozygous site, we determined haplotypes probabili-stically using PHASE software in DnaSP ver. 5.0 (Rozas *et al.*[42]). Haplotypes inferred by PHASE with a probability of >0.5 were highly consistent with those obtained by cloning method (Harrigan *et al.*[43]). To ensure accuracy, we used the haplotypes with a probability of >0.8 in subsequent analyses. In total, 1.14% of sequences were unable to be phased with a probability of >0.8.

### Sequencing analysis

For each species, we calculated the number of segrega-ting sites (*S*), average number of pairwise nucleotide differences per site (π; Nei, [44]), and *θ* (Watterson, [45]) for each locus. We estimated the minimum number of recombination events (*Rm*) within the 10 loci using the four-gamete test (Hudson & Kaplan, [46]). To evaluate the neutral evolution of each locus, Tajima’s *D* (Tajima, [23]) was estimated, and deviations from neutral expectations were evaluated by 10,000 coalescent simulations. In addition, D* and F* of (Fu & Li, [47]) were estimated for loci deviating from neutral expectations using coalescent simulations. These summary statistics were estimated using DnaSP ver. 5.0 (Rozas *et al.*[42]). In addition, the HKA test (Hudson *et al.*[48]) across loci was performed at the species level using the HKA program ( http://lifesci.rutgers.edu/$heylab/heylabsoftware.htm#HKA). This test is based on a prediction that follows from the neutral theory of molecular evolution, which states that loci exhibiting high rates of divergence between species will also exhibit high levels of variation within species.

### Phylogenetic and population structure analyses

Previous studies have indicated that the four species comprise monophyletic relationships in nuclear ribosomal and chloroplast DNA data (Mitsui *et al.*[21]). To estimate the phylogenetic relationships among the four species, we reconstructed a phylogenetic network using the NeighborNet method (Bryant & Moulton, [49]) implemented in SPLITTREE4 (Huson & Bryant, [50]) because relationships among populations may not conform to a treelike pattern due to shared ancestral polymorphisms and potential gene flow (Nordborg *et al.*[51]). Only this analysis was performed without phasing haplotypes, and heterozygous SNPs were coded according to IUPAC. We used the uncorrected-*P* distance as the metric, and ambiguous states were ignored in the analysis.

In addition, a Bayesian clustering analysis using STRUCTURE ver. 3.1 (Pritchard *et al.*[52]) was conducted to estimate the geographical structuring within species after determining the haplotypes for all samples. An admixture model was used, and the independence of allele frequencies among populations was assumed. The probability of assigning individuals into clusters was estimated using 5.0 × 10^5^ iterations, following 3.0 × 10^5^ iterations as the burn-in period. The number of clusters (*K*) was set from 1 to 20, and all runs were replicated 20 times to test the stability of the results. The most likely number of clusters was estimated according to the model value (Δ*K*) based on the second-order rate of change with respect to *K* of the likelihood function following the procedure described by Evanno *et al.*[24].

### Estimating population demographic parameters and fitting models of divergence

To estimate the population demographic history in the divergence of riparian and inland species, we employed the IM model (Nielsen & Wakeley, [53]; Hey & Nielsen, [8]) using the program IMa (Hey & Nielsen, [54]). The IM model uses a Markov chain Monte Carlo (MCMC) method to estimate the posterior probability densities of six demographic parameters: the divergence time (*t*), bidirectional migration rates (*m*_1_ and *m*_2_), and the effective population sizes of the ancestral (*θ*_A_) and descendent populations (*θ*_1_ and *θ*_2_). After several preliminary runs to optimize prior boundaries for the six parameters, we conducted runs for 3–5 × 10^6^ MCMC steps following a 300,000 burn-in period from which genealogies were saved every 100 steps. To ensure proper mixing, the run was performed using 20 independent chains and 10 swap attempts per step under Metropolis coupled with a geometric heating scheme of parameters *g1* = 0.05 and *g2* = 0.1. To check the convergence, plotted trend lines and the effective sample size (ESS) values were examined for each run. In addition, three independent runs for each data set were examined for consistency. An infinite site model of mutation was applied to all loci. The analyses were performed using the largest non-recombining block for each locus (see Table 1).

After all runs were converged under the full model, which estimates the six demographic parameters, we tested the fit of the data to simpler demographic models using the nested model approach in the L-Mode option of IMa. This test evaluates the presence or absence of gene flow between descendent populations and the population size differences among present and ancestral populations (Hey & Nielsen, [54]). We compared log-likelihoods of full and nested models in which one or both directions of gene flow were absent and present and ancestral populations were equal sizes.

Demographic parameters were scaled to the neutral mutation rate (*μ*). Mutation rates of nuclear genes for the studied samples were unknown. Thus, we calibrated the mutation rates of the GA2ox1 and CHS genes by comparing the sequences of perennial species in various taxa of Asteraceae because the studied species are perennials, although they can bloom and produce seeds within the year of germination (M. Mitsui, pers. obs.). A previous phylogenetic study by Kim *et al.*[55] estimated the splitting time among major Asteraceae clades and suggested that the tribes Cardueae and Mutisieae, as well as LALV (Lactuceae, Arctoteae, Liabeae, and Vernonieae) and Astroid, diverged *c*. 33–38 million years ago (mya). We calculated the average nucleotide divergence (*K*) in the sequences between the tribes Cardueae and Mutisieae and tribes LALV and Astroid using species from various taxonomic groups. The mutation rate per site per year (*μ*) was then calculated by *μ* = *K*/2 *T*, in which *T* = 35.4 mya was used for each of the GA2ox1 and CHS regions. The geometric mean of the mutation rates for GA2ox (6.79 × 10^–9^ substitutions/site/year) and CHS (8.26 × 10^–9^ substitutions/site/year) was estimated to be 7.49 × 10^–9^ substitutions/site/year, and this value was used to rescale the IMa parameter estimates.

## Competing interests

The authors declare that they have no competing interests.

## Authors’ contributions

YM designed the study, collected and assembled plant samples, carried out the molecular analyses and drafted the manuscript. HS participated in the overall design of the study, collected some of the samples, and contributed to drafting the manuscript. Both authors have read and approved the final manuscript.

## Supplementary Material

Additional file 1 **Table S1. **Haplotype compositions. The numbers represent the haplotypes of individuals at each locus. Click here for file

Additional file 2 **Table S2. **Results of neutrality tests for each of the 10 loci and each species. For Ainsliaea macroclinidioides, the southern populations were excluded in the analyses. Click here for file

Additional file 3 **Table S3. **Sampling localities. Sampling sites are shown in Figure 2. Click here for file
